# Premature Termination Codon in 5′ Region of Desmoplakin and Plakoglobin Genes May Escape Nonsense-Mediated Decay through the Reinitiation of Translation

**DOI:** 10.3390/ijms23020656

**Published:** 2022-01-07

**Authors:** Marta Vallverdú-Prats, Ramon Brugada, Mireia Alcalde

**Affiliations:** 1 Cardiovascular Genetics Center, IdIBGi, University of Girona, 17190 Girona, Spain; mvallverdu@gencardio.com; 2 Centro Investigación Biomédica en Red de Enfermedades Cardiovasculares (CIBERCV), 28029 Madrid, Spain; 3 Medical Science Department, School of Medicine, University of Girona, 17071 Girona, Spain; 4 Cardiology Service Hospital, University of Girona, 17007 Girona, Spain

**Keywords:** arrhythmogenic cardiomyopathy (ACM), CRISPR, genetics, desmosomal genes, HL1, premature termination codon (PTC), nonsense mediated decay (NMD), alternative translation initiation (ATLI)

## Abstract

Arrhythmogenic cardiomyopathy is a heritable heart disease associated with desmosomal mutations, especially premature termination codon (PTC) variants. It is known that PTC triggers the nonsense-mediated decay (NMD) mechanism. It is also accepted that PTC in the last exon escapes NMD; however, the mechanisms involving NMD escaping in 5′-PTC, such as reinitiation of translation, are less known. The main objective of the present study is to evaluate the likelihood that desmosomal genes carrying 5′-PTC will trigger reinitiation. HL1 cell lines were edited by CRISPR/Cas9 to generate isogenic clones carrying 5′-PTC for each of the five desmosomal genes. The genomic context of the ATG in-frame in the 5′ region of desmosomal genes was evaluated by in silico predictions. The expression levels of the edited genes were assessed by Western blot and real-time PCR. Our results indicate that the 5′-PTC in *PKP2, DSG2* and *DSC2* acts as a null allele with no expression, whereas in the *DSP* and *JUP* gene, N-truncated protein is expressed. In concordance with this, the genomic context of the 5′-region of *DSP* and *JUP* presents an ATG in-frame with an optimal context for the reinitiation of translation. Thus, 5′-PTC triggers NMD in the PKP2, DSG2* and *DSC2* genes, whereas it may escape NMD through the reinitiation of the translation in *DSP* and *JUP* genes, with no major effects on ACM-related gene expression.

## 1. Introduction

Arrhythmogenic cardiomyopathy (ACM) is a rare disorder characterised by progressive replacement of the myocardium by fibrofatty tissue. This myocyte disorganisation increases the risk of ventricular arrhythmias and sudden cardiac death [1,2]. Tissue substitution occurs predominantly in the right ventricle, but biventricular forms and predominant left ventricular involvement have also been reported [3,4]. The main genetic cause of ACM is mutations in desmosomal genes, representing around 50% of patients [5]. Desmosomes are cell unions that link the extracellular medium to intermediate filaments inside the cells to allow them to resist mechanical forces [6]. In cardiomyocytes, desmosomes are located in intercalated discs (IDs): highly specialised structures that also include adherents and gap junctions. These complexes connect cardiomyocytes electrically and mechanically in a functional syncytium [7]. Genes encoding desmosome proteins are plakophilin2 (*PKP2*), representing 10–45% of ACM cases; desmoplakin (*DSP*), responsible for 10–15% of cases; desmoglein2 (*DSG2*), responsible for 7–10% of cases; and desmocollin2 (*DSC2*) and plakoglobin (*JUP*), responsible for 1–2% of cases [5,8]. It is well known that a loss of function of desmosomal genes is associated with ACM [9]. In that sense, around 20% of described ACM-related mutations are frameshifts or nonsense mutations that cause premature termination codons (PTCs) in desmosomal genes [10,11].

It is widely accepted that the presence of PTCs in genes causes mRNA degradation via nonsense-mediated decay (NMD): a translation-dependent mRNA surveillance mechanism in eukaryotes that helps to maintain the quality of gene expression [12]. However, there are some cases in which mRNA with PTCs can escape from NMD, such as those located in the last exon [12]. Moreover, it has been described that the reinitiation of translation can occur in front of a PTC close to the start codon [13,14].

Normally, the translation is initiated when ribosome scanning finds the start codon. It is known that there has to be an optimal context around the initial ATG codon, and there are crucial positions with a high level of conservation within the Kozak motif that are essential for the initiation of translation: a purine at position −3 and a G at +4 [15]; in addition, A/C at −2 and no T on position +5 were also shown to be important (where the A of ATG is numbered +1) [14,16]. However, there are alternative ways to initiate translation that start in a non-canonical ATG [14]. This is the case of the reinitiation of translation that occurs when the first ORF is very short, and it starts in an alternative in-frame ATG after that first ORF [14,17]. In this way, translation reinitiation could allow the expression of an N-truncated protein by initiating translation at the in-frame methionines downstream of a PTC [14]. It is well accepted that the reinitiation of translation allows mRNA with PTCs to escape from degradation via NMD [13,18,19]. It has been observed that translation reinitiation is more efficient when the downstream in-frame methionine is located within 50 codons of the PTC when it is located in the first 10% of the coding sequence [13,20]. Moreover, there is evidence that the reinitiation of translation from ATG codons located over 160 codons downstream of the first initiation codon is significant [18]. Furthermore, it is well described that reinitiation is more efficient when the PTC is close to the canonical start codon [13].

Regarding the presence of PTCs in the 5′ region of desmosomal genes, it is uncertain whether those mutations are more susceptible to the activation of NMD or the reinitiation of translation. Moreover, it is not clear whether these five genes react similarly or differently in front of a PTC in the 5′ region. An important question to consider is what role these mechanisms might have in the overall ACM pathophysiology. It is described that the presence of a PTC in PKP2 causes it to lose function; thus, ACM is caused by haploinsufficiency [21]. However, it is unknown whether PTCs in other desmosomal genes have the same effect or not. It is important to describe whether ACM is caused by a null allele due to the mRNA degradation via NMD or by the presence of N-truncated protein caused by the reinitiation of translation in order to better understand the molecular mechanisms of the pathology, and also to classify the pathogenesis of new potential causal variants. There are many decisive factors in classifying rare variants, such as the mutation type, the location of the mutation and the affected gene [22]. Moreover, it is recommended to update the classification of rare variants associated with ACM due to its importance as a diagnostic criterion [23,24]. Understanding which mechanism is triggered by PTCs in the 5′ region of each desmosomal gene could allow a better comprehension of the pathology and more accurate prediction of the pathogenesis of new ACM-associated variants.

For this reason, the present study was aimed at evaluating the likelihood that desmosomal genes carrying PTCs at the 5′ region would trigger the reinitiation mechanism. We hypothesised that downstream reinitiation of translation would be triggered in some cases with optimal genomic context.

## 2. Results

### 2.1. PTC Clones and Genotype Distribution

Edited HL1 clones carrying frameshifts leading to a PTC within the 5′ gene sequence (fewer than 160 codons from natural ATG) in five desmosomal genes were selected for the study: *PKP2, DSP, DSG2, DSC2* and *JUP*. A total of 20 clones with at least one allele carrying PTC were obtained: four homozygous (HM) PKP2-PTC; four HM and two heterozygous (HT) DSP-PTC; four HM DSG2-PTC; two HM DSC2-PTC; and two HM and two HT-like JUP-PTC (Table 1).

**Figure 1:**
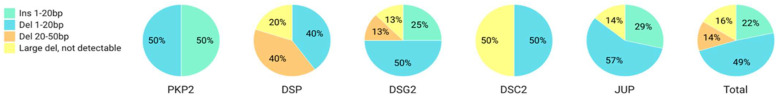
Prevalence of genetic variants introduced after repairing double strand break (DSB) in DNA caused by CRISPR-Cas9. Created with Datawrapper.

Our results indicate that 56.6% (17/30) of edited alleles presented a PTC in frame minus (−)2 and 43.3% (13/30) in frame −1 (Table 1). From all edited alleles, small insertions or deletions of fewer than 20 bp were the main genetic variation introduced in 67.5% of cases (25/37), predominantly deletions (45.94% of total, 17/37). Interestingly, larger insertions were not detected, while 16.2% (6/37) of PTCs were deletions of 20–50 bp and the remaining 16.2% (6/37) were large deletions (deletions affecting the whole exon, not detected by sequencing exon 1). Thus, deletions were the predominant variant identified, representing 78.3% (29/37) of total variants (Figure 1).

### 2.2. Genomic Context of 5′ Region for Reinitiation

Results from the analysis of the 5′ sequence of the five desmosomal genes predicted that the *DSP* and *JUP* sequences present alternative in-frame ATGs with high optimal sequence context for the reinitiation mechanism within the firsts 160 codons. On the other hand, *PKP2, DSG2* and *DSC2* genes present significantly fewer alternative in-frame ATGs, and none of them have high optimal genomic context for reinitiation (Table 2).

Similarly, in silico prediction suggests that *DSP* and *JUP* have in-frame ATGs with high optimal context susceptible to the reinitiation of translation. However, no in-frame ATGs present in *PKP2, DSG2,* or *DSC2* genes were predicted to be susceptible to the reinitiation of translation (Table 3).

### 2.3. Expression Levels of PTC Clones and Mechanisms Triggered

**Figure 2:**
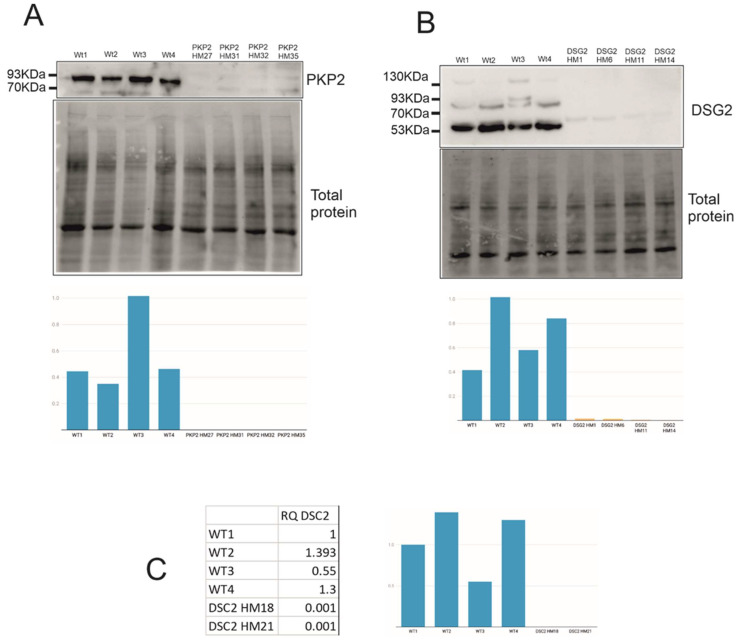
Undetectable levels of edited genes. (**A**) PKP2 protein expression of HM PKP2-PTC clones. (**B**) DSG2 protein expression of HM DSG2-PTC clones. (**C**) *DSC2* mRNA expression of two HM DSC2-PTC clones. Created with Datawrapper.

Expression levels of genes carrying PTC, either homozygous or heterozygous, were evaluated in all PTC clones. Protein expression levels were undetectable for all PKP2-PTC and DSG2-PTC edited clones (Figure 2A,B). Moreover, *DSC2* mRNA expression levels were null in HM DSC2-PTC clones (Figure 2C).

**Figure 3:**
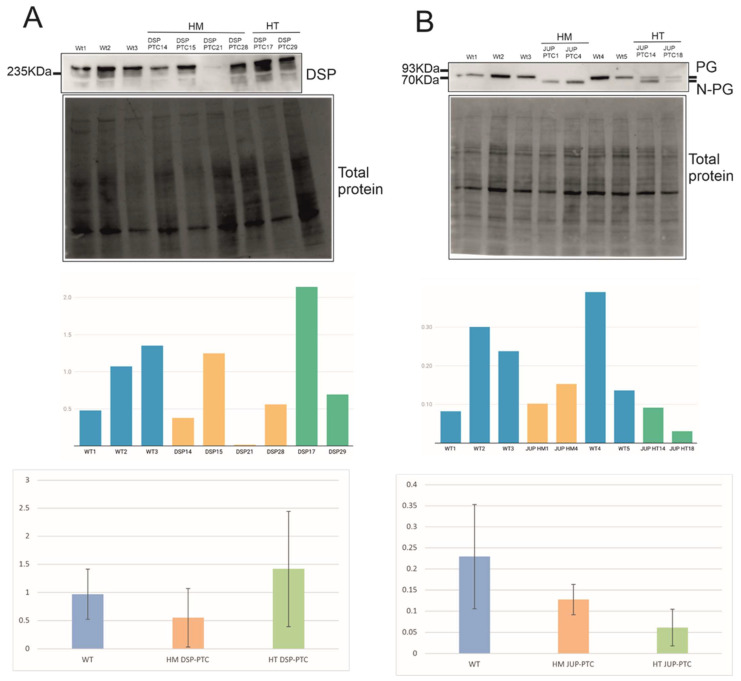
Reinitiation of translation. N-truncated protein expression for (**A**) DSP-PTC and (**B**) JUP-PTC clones.

On the other hand, DSP protein expression was detected in similar levels compared to WT clones in three of the four HM DSP-PTC clones (Figure 3A). The predicted size of WT DSP is 332 KDa (2883aa), whereas the expected size of N-truncated DSP would be around 307 KDa (2664aa), assuming that the translation starts in ATG positioned at c.514 with a high genomic context. Moreover, all HT DSP-PTC clones showed similar levels to WT, suggesting that either the reinitiation mechanism is triggered in the PTC allele or the WT allele can compensate the total expression of DSP.

Finally, PG protein expression in the two HM JUP-PTC clones suggests that the reinitiation mechanism was triggered, translating an N-truncated PG using a downstream alternative in-frame ATG (Figure 3B). N-truncated PG was clearly observed in these clones with a lower weight than PG-WT, compatible with the predicted size of N-truncated PG using a downstream ATG (the predicted size of WT is 81 KDa (745aa) and that of N-truncated PG is around 67 KDa (619aa), assuming that the translation starts in ATG positioned at c.248 with a high genomic context). Interestingly, the two HT JUP-PTC clones showed a double band corresponding to the WT size and the N-truncated PG size, suggesting that the PTC allele would also trigger the reinitiation mechanism in HT genotypes. All PG clones expressed similar levels of the protein compared with WT (Figure 3B).

mRNA expression levels of *PKP2, DSG2, DSP* and *JUP* edited clones are shown in Supplementary Figure S1.

Taking all the data together, the reinitiation mechanism was triggered only in *DSP* and *JUP* genes, which were those predicted to present close downstream in-frame ATGs with optimal sequence context likely to be susceptible to the reinitiation of translation. All PTC alleles in JUP-PTC clones, in both HM and HT genotypes, produced an N-truncated PG (Figure 3B). However, this mechanism occurred in only 75% of HM in DSP-PTC clones, suggesting that reinitiation is not consistent between individuals, although it is over time. The expression of these PTC alleles does not depend on the distance between PTC and the canonical start in our study. In the *DSP* gene, this distance corresponds to 17 codons in −1 frame, representing 25% (2/8) of the total edited alleles, and 75 codons in −2 frame, representing 75% (6/8) of alleles. In the *JUP* gene, the distance corresponds to 37 codons between PTC and the canonical start in −1 frame, representing 60% (3/5), and 44 codons in −2 frame, representing 40% (2/5) of the total edited alleles (Table 1).

### 2.4. Effect of N-Truncated DSP and JUP on ACM-Related Gene Expression

**Figure 4:**
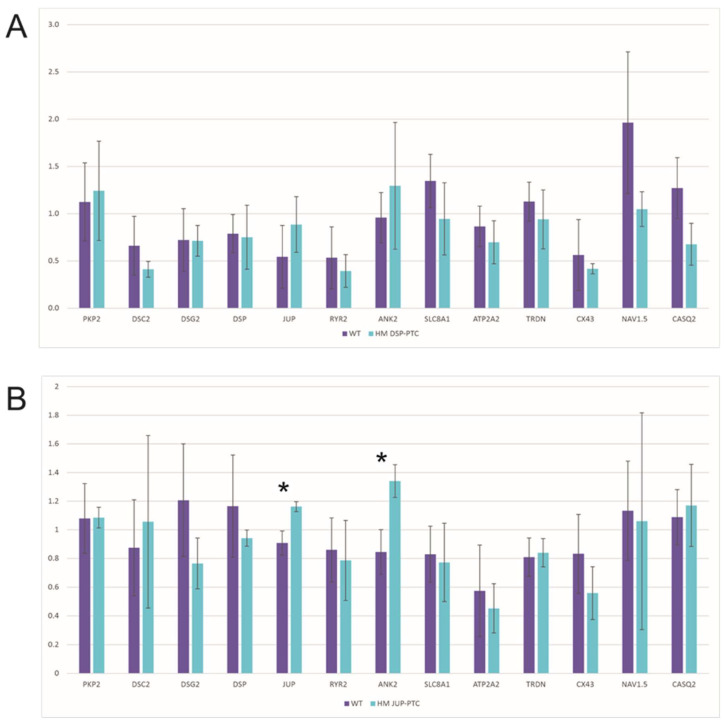
mRNA expression level of desmosomal, calcium handling and connexome genes in (**A**) HM DSP-PTC and (**B**) HM JUP-PTC clones. * Marks those gene expression levels that were significantly different between WT and edited clones.

Expression levels of genes involved in ACM, such as desmosome, calcium handling and connexome genes, are slightly altered in HM JUP-PTC clones that express the PG N-truncated protein (Figure 4B). Even though these clones showed similar levels of expression of the truncated protein compared with WT, HM JUP-PTC presented significant upregulation in *JUP* and *ANK2* genes (Supplementary Tables S1 and S2).

On the other hand, the three HM DSP-PTC clones that expressed the DSP N-truncated protein did not show any molecular alterations in the studied genes involved in ACM (Figure 4A, Supplementary Tables S1 and S3). However, only one HM DSP-PTC clone had no detectable levels of DSP protein, suggesting important differential expression at the RNA level compared with WT, but statistical analysis was not possible because only one clone was available. Nevertheless, it is important to note that *DSC2* expression was nearly null in this clone, RQ = 0.001 (Supplementary Table S3).

## 3. Discussion

In ACM, loss of function due to NMD and triggered by PTC codon variants has been widely supported for all five desmosomal genes [9,21,26,27,28,29]. The position of a truncating mutation can govern the resulting phenotype, as mutations in the last exon can evade NMD [12]. In the present study, we investigated the susceptibility to NMD of PTC located at the end 5′ sequence for all five desmosomal genes associated with ACM (*PKP2, DSP, DSG2, DSC2* and *JUP*) and whether they might trigger downstream reinitiation of translation within the optimal genomic context. Concretely, we investigated whether a putative downstream in-frame ATG after a PTC would be susceptible to the reinitiation of translation. Our results show undetectable levels of PKP2, DSG2 protein and *DSC2* mRNA in 100% of clones, suggesting that PTC-mRNA or protein were targeted and degraded by a quality control mechanism when carrying a PTC within the first 160 codons. Interestingly, although *PKP2, DSG2* and *DSC2* genes presented alternative ATG in-frame within the region <160 codons from the natural ATG, none with highly optimal genomic context was found. These results suggest that the optimal genomic context of ATG in-frame may be key for the reinitiation of translation.

On the other hand, both *DSP* and *JUP* presented alternative in-frame ATG close to the initial codon with an optimal context. In the *DSP* sequence, the ATG in-frame with high context accomplished the −3 position of Kozak motif, but not the +4 position. However, it presented C at −2 and no T on position +5, which was also described as being important [14,16]. The *JUP* sequence around the alternative ATG in-frame predicted to present high genomic context accomplished both positions of Kozak motif. Moreover, it presented no T on position +5, and no A/C at −2. In concordance, *DSP* and *JUP* showed expression of N-truncated protein when containing PTC by escaping NMD, probably due to the reinitiation mechanism. Concretely, the *JUP* gene expressed N-truncated PG protein in 100% of cases, and DSP in 75% of homozygous clones, suggesting that other factors might be involved in triggering this mechanism, with consistency over time for each clone. Previous studies showed that the distinguishing feature of most of these mutations evading NMD was proximity to the initiation codon. Evasion of NMD has been proposed to be mediated by the reinitiation of translation [30,31,32] or by physical proximity to the native ATG initiator codon [33]. However, there is still no knowledge about the possible role of the other allele in the NMD evasion mechanism, i.e., whether carrying other PTCs in trans- or wild-type alleles might have an influence.

Concerning mRNA expression levels of edited clones, results show that *DSP* and *JUP* clones express similar PTC-mRNA levels to WTs clones (Figure S1), in concordance with protein levels (Figure 3). Moreover, *PKP2* clones present a significant downregulation both in PTC-mRNA (Figure S1) and PTC-protein levels (Figure 2), probably due to NMD. On the other hand, two of the four *DSG2* clones present similar PTC-mRNA levels compared with WTs (Figure S1), but no protein expression (Figure 2). This discordance may be explained by the intrinsic dynamic properties of NMD that could be repressed under cellular stress [34]. However, proteolytic machinery could act when NMD misses defective mRNAs [35]. What is important here is that we have proven that *DSG2* edited clones did not experience the reinitiation of translation but NMD or protein degradation was triggered by PTC in the 5′ region. Finally, we were only able to detect PTC-mRNA levels, but no protein levels, in *DSC2* edited clones which were proven to be null (Figure 2), probably also due to NMD.

Regarding the role of DSP N-truncated protein in ACM pathogenesis, our results show that HM DSP-PTC clones had no alterations of the total RNA expression levels in ACM-related genes, suggesting that these PTCs generating N-truncated *DSP* may not be loss-of-function variants and may not be associated with severe changes in genes involved in the molecular pathomechanism of ACM. First, the *DSP* loss-of-function pathomechanism is supported by several mouse models [36,37,38], and PTC variants represent a relatively high amount (5–10%) of pathogenic truncating *DSP* mutations associated with the ACM phenotype, preceded only by PTC in the *PKP2* gene. The *PKP2* gene presents PTC variants as the most prevalent type of mutation [39,40]. The results of the present study suggest that the expression of N-truncated protein in *DSP* clones can escape NMD and the molecular alterations associated with ACM. In this sense, some studies have shown that PTC close to the initial codon may be associated with a milder phenotype of diseases due to the reinitiation of translation [13,14,41,42,43,44]. To date, there are no studies exploring a similar effect of translation reinitiation in desmosomal genes carrying PTCs at the 5′ region and their association with the ACM phenotype and disease severity. Interestingly, the DSP clone, which did not trigger the reinitiation mechanism and therefore acted as a null clone, showed a different pattern in the gene expression profile in ACM-related genes, such as downregulation of *DSC2*, supporting the idea that N-truncated DSP protein might be associated with milder cellular phenotype or no phenotype at all. However, more studies should be performed to further investigate molecular alterations when DSP is not expressed at the protein level.

On the other hand, PTC in the 5′ region of *JUP* showed reinitiation in 100% of cases synthesising N-truncated instead. These results suggest that the N-ter aminoacids of PG might be important in maintaining the normal RNA levels of *ANK2*, but further studies with larger amounts of clones would be needed to confirm that. It is known that the N-ter of PG protein is a conserved region between human and mouse [45], and it has been described as participating in binding β-catenin and promoting desmoglein-2 association [45,46,47]. However, it is still unknown whether alterations in PG can affect *ANK2* expression, and more studies should be performed.

Taking all of the above into account, this study suggests that when reinitiation is triggered, the molecular phenotype might be milder than or not associated with the ACM pathomechanism. In this sense, clinical evidence, such as pathogenic variants upstream of the new alternative codon, also points to there being no critical biological relevance of the N-terminal region in relation to the ACM phenotype. The number of PTC mutations described in the first 160 codons of *DSP* and *JUP* genes in the general population and ACM patients supports the supposition that there might not be an association of N-ter loss and ACM phenotype in *DSP* and *JUP*. In the *DSP* gene, only two variants causing PTC have been described in ACM patients [11] and five in the general population [48]. Patients carrying DSP: p.R160X had no cardiac dysfunction at the age of examination (8 months old) [49]. Similarly, in the *JUP* gene, only one variant has been described in ACM patients [11] and three in the general population [48]. *JUP* p.S24X has been identified in a patients with cutaneous disease, but no symptoms of cardiomyopathy in children were reported [50]. In contrast, in the *PKP2* gene, which is the main gene in ACM, according to our results, is not able to trigger reinitiation, showing 17 different 5′-PTC variants in the first 160 codons in ACM patients [11] and only six in the general population [48]. It is accepted that *PKP2* haploinsufficiency causes ACM [21], and this may suggest that PTCs in the first 160 codons of *PKP2* are pathogenic due to the lack of translation reinitiation mechanism.

This raises a concern regarding how to classify 5′-PTC variants in terms of pathogenicity in *DSP* and *JUP* genes. For the interpretation of loss-of-function variants based on ACMG guidelines [22], it is recommended to apply very strong or strong criteria to PTCs in all desmosomal genes [9], regardless of position. Currently, the criterion applied for those PTC variants located in the last exon of the gene is only moderate, taking into account that they are likely to escape NMD decay. Our results suggest not only that last exon PTCs can avoid NMD machinery, but also that end 5′ PTCs in *DSP* and *JUP* genes can synthetise N-truncated proteins with little to no great impact on the expression of ACM-related genes.

Regarding the limitations of the study, one of the most important restrictions is that the number and distribution of genotypes are different for each gene, since it was not possible to predict what would be the final outcome of editing. Second, the distance between the canonical start codon and PTC is not identical among desmosomal genes because of the different nature of each sequence, even though, as is known, the reinitiation of translation is more efficient when this distance is shorter [13]. Third, despite the high similarity between human and mouse desmosomal sequences, the results should be interpreted with caution when applied to human genes, since the two are not identical. Further experimental evidence from human samples would be needed to validate this mechanism in humans. On the other hand, although HM DSC2-PTC showed nearly no mRNA expression of the edited clones suggesting NMD, ideally this also should be checked by immunoblot. Unfortunately, none of the commercial antibodies worked in our cellular model and conditions. Finally, N-truncated DSP was presumably expressed in HM DSP-PTC with around 307 KDa (2664 aa), assuming that the translation starts in ATG positioned at c.514 with high genomic context, only 25 KDa less than full length DSP (7.5% of the total weight), and it was not possible to differentiate N-truncated protein in the electrophoresis gel. Moreover, N-term anti-DSP antibody targeting only full-length DSP and not N-truncated DSP is not available in the market. Finally, further studies to evaluate N-truncated DSP and PG will be needed to understand whether they have full functionality or might be involved in the ACM cellular phenotype and other PTC mutations along the whole sequence of desmosomal genes.

## 4. Materials and Methods

*sgRNA design and cloning into Cas9px458 vector*. The Benchling Web tool (Biology Software, 2018; retrieved from https://benchling.com; accessed on 2 March 2018) was used to design the sgRNAs of the 5 desmosomal genes at the first 160 codons of the sequences for editing by CRISPR/Cas9. sgRNAs with high scores and low off-targets were selected: *PKP2* (GTATGTCTACAAGCTACACG, FW); *DSP* (CCACCCGCGGATCAACACGC, FW); *DSG2* (TGGCGCGGAGCCCGGGTGAC, FW); *DSC2* (GCTGTGGGATCTATGCGCTCC, FW); *JUP* (CCTTGATGGGCTGCTCAATA, RV). px458 vector (plasmid #48138, addgene, Teddington, UK), which encodes Cas9 WT, was digested by BbsI-HF (R3539S, New England BioLabs, Ipswich, MA, USA) at 37 °C overnight and ligated by T4 DNA ligase (M0202L, New England BioLabs, Ipswich, MA, USA) for 1 h at RT with the sgRNA previously annealed (sense and antisense). Annealing of sgRNAs was performed with T4 PNK (M0201S, New England BioLabs, Ipswich, MA, USA) using the following thermocycler program: 30′ at 37 °C, 5′ at 95 °C and 94 to 25 °C going down 1° per 12′′. DH5alpha competent cells (18265-017, Invitrogen, Waltham, MA, USA) were transformed with sgRNA-px458 vector during 30′ in ice and 45′′ at 42 °C. DNA was extracted using the Plasmid Midi Kit (12143, Qiagen, Hilden, Germany).

*HL-1 cell culture and electroporation.* HL1 cells were cultured as described previously [51] at 37 °C under 5% CO_2_ in fibronectin–gelatin coated slides in supplemented Claycomb medium (51800 C–500 ML, Sigma, St Louis, MO, USA) with 10% fetal bovine serum (10270106, GIBCO, Waltham, MA, USA), 100 U/mL penicillin, 100 μg/mL streptomycin (P4333-100 ML, Sigma, St Louis, MO, USA), 2 mM L-glutamine (35050061, Thermo, Waltham, MA, USA), 10 μM norepinephrine (A9512, Sigma, St Louis, MO, USA) and 0.3 mM ascorbic acid (A7631, Sigma, St Louis, MO, USA). Plasmids were nucleofected into HL-1 in suspension by using the Amaxa Cell Line Nucleofector Kit V (VCA-1003, Lonza, Basel, Switzerland); 10^6^ cells per condition were transfected by adding 4 ug of the vector. After that, cells were seeded into 24-well plates, and after 48 h, they were diluted by seeding 10,000 cells in a P100 and 5000 cells into a 6-well plate. When colonies started growing, they were picked and seeded into a 24-well plate. Cells were expanded and frozen in a vial (with claycomb medium and 10% DMSO (D2650-5X5ML, Sigma, St Louis, MO, USA)) and a pellet to extract gDNA.

*gDNA extraction and Sanger sequencing*. To extract gDNA of the HL1 clones, QuickExtract (QE09050, Lucigen, Middleton, WI, USA) was used. For this process, 20 μL of the reagent was added to each pellet and vortexed for 13 s. Samples were incubated at 65 °C for 6 min, vortexed for 15 s and incubated at 98 °C for 2 min. Primers, PCR conditions and kits used are listed in Supplementary Table S4. After that, ExoZap cleaning (7200100-1000, Ampliqon, Odense, Denmark) and BigDye reaction (4336911, Applied Biosystems, Waltham, MA, USA) were performed. DNA precipitation was done by adding sodium acetate and ethanol 70% and diluted in formamide. Samples were sequenced using a 3500 Genetic Analyzer (Applied Biosystems, Waltham, MA, USA). Sequencing Analysis Software 7 was used to analyse the sequences.

*RNA extraction and real-time PCR (rtPCR)*. Total RNA was purified using the RNeasy Mini Kit (74106, Qiagen, Hilden, Germany) according to the manufacturer’s instructions. Prior reverse transcription reaction was performed with an additional step of DNase I treatment and with gDNA Wipeout buffer. Reverse transcription reactions of RNA were done using the QuantiTect Reverse Transcription Kit (205313, Qiagen, Hilden, Germany). For each 20 μL reverse transcription reaction, 1 ug of total RNA was mixed with 4 uL of RT buffer, 1 uL of primer mix, 1 μL of reverse transcriptase in nuclease-free water. cDNA was analysed in real-time PCR reactions using KAPA SYBER FAST Universal Kit (KK4602, KAPA biosystems, St Louis, MO, USA). RPLP0 was used as housekeeping and all data were analysed using the QuantStudio™ Real-Time PCR System and Cloud Software (Thermo Fisher, Waltham, MA, USA). The obtained results were analysed statistically by performing a bilateral Student’s *t*-test using SPSS.

*Protein extraction and Western blot*. Total protein was extracted by lysing the cells with 1% SDS, incubating at 95 °C for 15 min and vortexing for 15 min more. Protein samples were quantified using a Pierce BCA Protein Assay Kit (23225, Thermo Scientific, Waltham, MA, USA) and run in a 10% acrylamide stain-free gel (1610183, Bio-Rad, Hercules, CA, USA) using the BlueStar Prestained Protein Marker Plus (MWPO4, Nippon Genetics, Düren, Germany) for 30 min at 80 V and 1 h at 160 V. Proteins were transferred from gel to PVDF membranes (10600023, GE Healthcare Life Sciences, Boston, MA, USA) for 2 h at 80 V and 4 °C. Stain-free gels were exposed to UV light before protein transfer and then membrane to activate the trihalo compound that reacts with tryptophan residues, allowing rapid fluorescent detection of total protein to normalise the results. Membranes were blocked with PBST + 5% non-fat milk for 1 h at RT and incubated with primary antibody anti-desmoplakin 1/2 (2722-5204, Bio-Rad, Hercules, CA, USA) at 1:500, anti-plakoglobin (13-8500, Invitrogen, Waltham, MA, USA) at 1:1000, anti-plakophilin2 (ab189323, Abcam, Cambridge, UK) at 1:250, anti-desmoglein2 (ab150372, Abcam, Cambridge, UK) at 1:3000 o/n at 4 °C. After several PBS washes, the membranes were incubated with peroxidase-conjugated anti-rabbit antibody (111-035-003, Jackson ImmunoResearch, West Grove, PA, USA) or anti-mouse antibody (115-035-003, Jackson ImmunoResearch, West Grove, PA, USA) and anti-goat (705-035-003, Jackson ImmunoResearch, West Grove, PA, USA) at a 1:10,000 dilution for 1 h at room temperature. Chemiluminescent signal was obtained with clarity substrate (1705061, Bio-Rad, Hercules, CA, USA) and detected using the ChemiDoc MP imaging system. Expression levels were quantified by Image Lab software using total protein of the stain-free gels to normalise [52]. The obtained results were analysed statistically by performing a bilateral Student’s *t*-test using SPSS.

*Prediction of the score of alternative ATG in the alternative initiation translation*. altORFev [25] was used to perform the prediction of translation reinitiation in the 5′ region of the five desmosomal genes. The advanced mode (http://wwwmgs.bionet.nsc.ru/AUGWeb/; accessed on 24 September 2021) was used by pasting the first 160 codons of the coding sequence (without UTRs) and by changing one parameter, the min ORF size. Generally, 30 (codons) is the default value, but in the present study, it was changed by 120 codons because it is the larger distance between the canonical ATG and the PTC of the clones (Table 1); in this way, all alleles were susceptible to the reinitation of translation. In-frame ATGs with high, moderate and weak contexts were classified using the criteria from the altORFev tool [25].

## 5. Conclusions

Our study suggests that the reinitiation of translation may be an important mechanism in evading the NMD machinery in *DSP* and *JUP* genes when carrying PTC variants in the 5′ region due to an optimal genomic context of a non-canonical ATG in-frame. In contrast, PTC variants in the first 160 codons in *PKP2, DSG2* and *DSC2* genes are more likely to trigger the NMD mechanism. Moreover, our data also suggest that molecular alterations associated with the ACM pathomechanism may be milder or non-detectable for N-truncated PG and N-truncated DSP. These data, together with the lower ratio of pathogenic 5′-PTC described in *DSP* and *JUP* versus *PKP2* (which do not show a reinitiation mechanism), also suggest the possibility that the reinitiation mechanism in PG and DSP may also occur in humans, and N-truncated protein synthesis may not be associated with severe ACM disease phenotype. Thus, these findings suggest that PTC variants in 5′ region have different pathogenicity depending on which desmosomal gene is involved. Taking into account that genetics counts as an ACM diagnostic criterion, it would be important to perform more studies in that direction.

## Figures and Tables

**Table 1:** CRISPR edited desmosomal gene clones.

Desmosomal Gene	Clones		Variants	New Frames	PTC Positions (Codons)
*PKP2*	27	A1	c.325ins1	−2	93
		A2	c.324del2	−2	93
	31	A1	c.326ins1	−2	93
		A2	c.322del2	−2	93
	32	A1	c.325ins1	−2	93
		A2	c.324del1	−1	115
	35	A1	c.325ins1	−2	93
		A2	c.325del4	−1	115
*DSP*	28	A1	c.329del2	−2	75
		A2	c.331del5	−2	75
	21	A1	c.332del2	−2	75
		A2	c.332del4	−1	17
	14	A1	c.324del29	−2	75
		A2	NI	-	-
	15	A1	c.326del31	−1	17
		A2	NI	-	-
	17	A1	WT	0	-
		A2	c.324del26	−2	75
	29	A1	WT	0	-
		A2	c.314del32	−2	75
*DSG2*	1	A1	c.226del4	−1	31
		A2	c.229ins1	−2	38
	6	A1	c.220del 30 + ins7	−2	38
		A2	NI	-	-
	11	A1	c.220del7	−1	31
		A2	c.228del1	−1	31
	14	A1	c.221del10	−1	31
		A2	c.229ins1	−2	38
*DSC2*	18	A1	c.324del4	−1	20
		A2	NI		
	21	A1	c.325del1	−1	20
		A2	NI		
*JUP*	1	A1	c.135del13	−1	37
		A2	c.142del4	−1	37
	4	A1	c.142ins1	−2	44
		A2	NI	-	-
	14	A1	c.136del6	0	-
		A2	c.142ins1	−2	44
	18	A1	WT	0	-
		A2	c.135del10	−1	37

**Table 2:** Sequences of 5′ UTR (lowercase) and the first 160 codons (alternating white and grey; 480 base pairs) of 5 desmosomal mouse genes. Exons are shown in alternating black and blue text. The underlined sequence is the hybridisation region of sgRNA used to edit desmosomal genes in the HL1 cell line by CRISPR/Cas9. ATG codons in-frame are in green, blue or red depending on genomic context for translation reinitiation. ATGs with high optimal context (ANNATGN, GNNATGG) are highlighted in green, moderate context (CNNATGG, UNNATGG, GNNATGH) in blue and weak context (YNNAUGH) in red, where Y = C or U, H = not G (http://wwwmgs.bionet.nsc.ru/AUGWeb/; accessed on 24 September 2021) [25].

***PKP2***	tctgcggctttgcgggcaggtcctggcagtccctcctggtcactccgccgacgcgATGGCCGTCCCCGGCTCACTGGCCGAGTGTGGCTACATCCGGACTGTGCTGGGCCAGCAGATCCTGGGTCACCTGGACAGCTCCAGCCTGGCCTTGCCCTCCGAGGCCAGACTGAGGCTGGCCGGCAGCAGCGGCCGCGGCGACCCGGCGGCCCGGAGCCAGCGGATCCAGGAGCAGGTGCAGCAGACCCTGGCCCGCCGGGGCCGGAGCTCTGCGGTCAGCGGGAACCTTCACCGAACCAGCAGTGTCCCTGAGTATGTCTACAAGCTACACGTGGTTGAGAATGACTTTGTTGGACGGCAGTCACCTGTCACTAGGGACTATGACATGCTTAAGGCTGGAATGACTGCCACTTATGGAAGTCGCTGGGGGAGAGCAGCAGCACAGTACAGTTCCCAGAAGTCAGTGGAGGAGAGATCCTGGAGGCAGCCTCTGAGGAGACTTGAGATTTCCCCAGATAGCAGCCCGGAGAGAGCCCAC
***DSP***	gaccaggtgtggcctgggcgccgggtgccagcggggaggagactcgcaccgcctcgaccaacaccaacacccaggcgcgacccagctcctctgagccctcgctgccctccgagccacagctccactccggttcccgcgcctagccagtcgccgtccccgtctccgccctgctggagcgctgagccctcgccagtcctccgcgttccgcgctcctctcccggagtccctcgcgtgctccgaggcgacgcctcgcgtatgccgcggcgctgagcggctctcttgagtgaccgcagacATGAGCTGCAACGGCGGCTCCCACCCGCGGATCAACACGCTGGGTCGCATGACCCGCGCGGAGTCCGGCCCGGACCTGCGCTACGAGATGACCTACAGCGGTGGCGGCGGCGGGGGCGGCGGGGGCGGCGGCGGGGGCACCAGCAGGACGTTCTACTCCCACTCCCGGCGCTGCACCGTCAACGACCAGAACTCCGACGGCTACTGTCAAACCGGCACCATGTCTAGACATCAGAATCAGAACACCATCCAAGAAATGCTGCAAAATTGCTCAGACTGTCTGATGCGGGCGGAGCTGATCGCGCAGCCGGAACTGAAATTCGGAGAAGGGATGCAGCTGGCATGGAACCGAGAGCTGGATGAGTATTTTACACAAGCCAACGATCAGATGGAAATCATAGACGGCTTGATCCGAGAGATGAGGCAGATGGGCCAGCCCTGTGATGCGTATCAGAAAAGACTGCTTCAGCTCCAGGAACAA
***DSG2***	ctgcctcgtacctccccgcggagcgaacacattcccctcctccatctaggctgtggcccggcccaaggctaccctttctgacccgggcacacctggaaccgcaccccgggtcccgcagagtcagagaagggcggccccgggagggacctgcccaggaggatccgcagggcgccggcgaggcccggaggcgagggcgcggcggatcgaggcgATGGCGCGGAGCCCGGGTGACCGGTGCGCCCTGCTGCTGCTGGTGCAGCTGCTGGCGGTGGTCTGCTTGGACTTTGGAAACGGACTTCACTTAGAGGTCTTCAGCCCAAGAAATGAAGGCAAACCGTTCCCTAAGCACACTCACTTGGTTCGTCAAAAGAGGGCCTGGATCACTGCCCCTGTGGCTCTGCGGGAGGGCGAAGACCTGTCCAGAAAGAACCCGATTGCCAAGATACACTCTGACCTTGCAGAAGAAAAAGGGATAAAAATCACGTACAAGTACACTGGGAAGGGAATTACAGAACCGCCTTTCGGCATATTCGTCTTTGATAGAAACACAGGAGAACTGAACATCACTAGCATTCTTGACCGGGAAGAAACACCATATTTTCTGCTGACAGGCTATGCATTGGACTCCAGAGGAAACAACCTGGAAAAGCCCTTGGAACTACGCATCAAAGTTCTGGACATCAATGACAAC
***DSC2***	ggggggtgacaagggacatataggtggcctctgctggtgagaaatacctagtacaggtgaaagggtggcggccagagggagttcccaccggttgaattcttaaagtcatgaagactcaagaaaaataacaaggagtggccgattcgagtcttttggacttgcccagagctccaccctcggacagaggaaaagcccctggggacccaggcgggagcatcagacaagcgcgagaaaagcgcctgtgtgcgcgctccacctctgcgcagcgggtgcggggcggtgacctgtcccttgagctggccATGGCGGCTGTGGGATCTATGCGCTCCGGGAGCCCTGCCTTCGGCCTGGGACACCTGTTGACCCTTGCGATCCTTGCACTTGCCTCTGATGCCTGTAAAGAAGTCGTCCTCCAGGTCCCCTCTGAACTACCTGCCGAGAAATTTGTTGGCAGAGTGAACCTGATGGACTGCCTTAAATCAGCAGACATAGTTCATCTGAGTGATCCTGACTTCCAAGTCTTAGAAGATGGTTCTGTGTACACAACCAGTTCTGTTGTTTTGTCCTCGGGGCAAAGAAGCTTTACTATATGGCTTTTTAGCACAGACAGCCAAGAAGAAAGGGAGATATCTGTCCATTTAGAGGGCCCAGTAGAGGTACTAAATAAAAGACCGCATACAGAGAAGGTTCTCAGCCGTGCCAAGAGAAGATGGGCTCCTATCCCTTGTTCCATGCTAGAGAATTCATTGGGTCCCTTCCCACTTTTCCTTCAACAGATCCAG
***JUP***	gccagagtccggagcagccgccgcccgagtgcgccgagctcagttcgctgcccgcgccggctccctcccggccagacccgaccccgattcggctcagcccggctccacgctcagcagccaccATGGAGGTGATGAACCTTATTGAGCAGCCCATCAAGGTGACAGAGTGGCAACAGACATACACCTACGACTCGGGCATCCACTCCGGCGTCAATACCTGTGTGCCCTCTGTAAGCAGCAAGGGCATCATGGATGAGGATGATGCCTGCGGCAGACAGTACACACTCAAGAAGACTACCACCTATACACAAGGGGTGCCACAGAACCAAGGTGACCTGGAATACCAGATGTCCACAACGGCCAGAGCCAAGCGGGTGCGGGAGGCCATGTGTCCAGGGGTCTCAGGCGAGGACAGTTCTCTACTGCTGGCCACCCAGGTGGAGGGGCAGACAACCAATCTGCAGCGCCTGGCCGAACCATCCCAGTTGCTCAAGTCGGCCATCGTCCATCTCATCAACTACCAGGATGATGCAGAGCTGGCCACCCGGGCTCTGCCTGAGCTCACCAAGCTGCTCAACGATGAGGACCCGGTAGTGGTGACC

**Table 3:** In silico prediction of context for translation reinitation of ATGs within the first 160 codons of 5 desmosomal genes: *PKP2, DSP, DSG2, DSC2* and *JUP*. Prediction takes into account all ATGs, in-frame and not. ORFs that start with ATG in-frame and have high genomic context are highlighted in orange.

	Name	In-Frame	Remark	Context	Mechanism	Position
*PKP2*	Orf1	Yes	Null, canonical ATG	W	Linear scanning	0
	Orf2	No	AltORF2; predicted translation level: moderate	H	Reinitiation starter	256
*DSP*	Orf1	Yes	Null, canonical ATG	W	Linear scanning	0
	Orf2	Yes	AltORF2; predicted translation level: weak	W	Leaky scanning	48
	Orf3	Yes	AltORF3; predicted translation level: moderate	M	Leaky scanning	87
	Orf4	Yes	AltORF4; predicted translation level: moderate	H	Reinitiation starter	219
*DSG2*	Orf1	Yes	Null, canonical ATG	W	Linear scanning	0
	Orf2	No	AltORF2; predicted translation level: moderate	M	Leaky scanning	112
	Orf3	No	AltORF3; predicted translation level: moderate	M	Leaky scanning	403
	Orf4	No	AltORF4; predicted translation level: weak	W	Leaky scanning	472
*DSC2*	Orf1	Yes	Null, canonical ATG	W	Linear scanning	0
	Orf2	Yes	AltORF2; predicted translation level: weak	W	Leaky scanning	18
	Orf3	No	AltORF3; predicted translation level: weak	W	Leaky scanning	88
	Orf4	Yes	AltORF4; predicted translation level: moderate	M	Leaky scanning	162
	Orf5	No	AltORF5; predicted translation level: moderate	H	Reinitiation starter	226
*JUP*	Orf1	Yes	Null, canonical ATG	W	Linear scanning	0
	Orf2	Yes	AltORF2; predicted translation level: moderate	M	Leaky scanning	9
	Orf3	Yes	AltORF3; predicted translation level: moderate	H	Reinitiation starter	126

## Supplementary Materials

The following are available online at https://www.mdpi.com/article/10.3390/ijms23020656/s1.
