# Supplementary material for: Premature Termination Codon in 5′ Region of Desmoplakin and Plakoglobin Genes May Escape Nonsense-Mediated Decay through the Reinitiation of Translation
Source: Int J Mol Sci. 2022 Jan 7;23(2):656. doi: 10.3390/ijms23020656 (PMC8775493; doi:10.3390/ijms23020656)
Supplement: Supplementary file 1 [file ijms-23-00656-s001.zip › ijms-1532302-supplementary.pdf]

Supplementary Material

A

	RQ PKP2
WT1	0.800
WT2	0.845
WT3	0.785
WT4	1.000
PKP2 HM27	0.126
PKP2 HM31	0.193
PKP2 HM32	0.164
PKP2 HM35	0.094

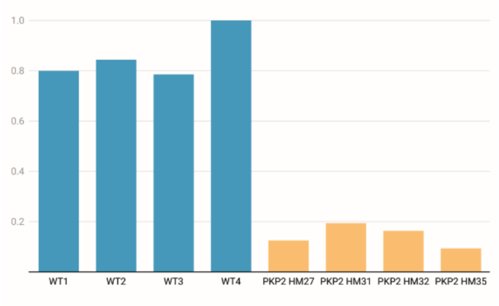

B

	RQ DSG2
WT1	1
WT2	0.963
WT3	0.812
WT4	1.405
DSG2 HM1	0.914
DSG2 HM6	0.334
DSG2 HM11	0.419
DSG2 HM14	1.863

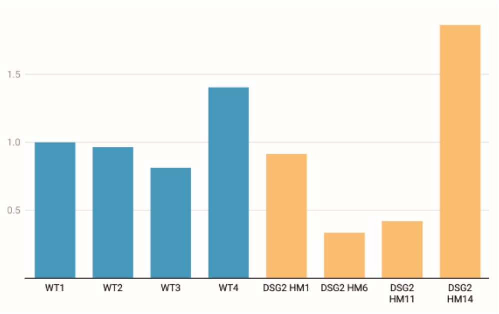

C

	RQ DSP
WT1	1
WT2	0.663
WT3	0.576
WT4	0.919
DSP HM14	0.381
DSP HM15	1.046
DSP HM21	0.370
DSP HM28	0.825
DSP HT17	1.060
DSP HT29	0.882

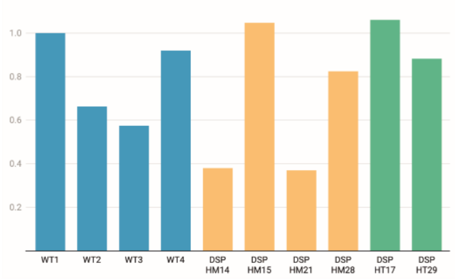

D

	RQ JUP
WT1	1.000
WT2	0.800
WT3	0.935
WT4	0.899
JUP HM1	1.187
JUP HM4	1.138
JUP HT14	0.900
JUP HT18	0.688

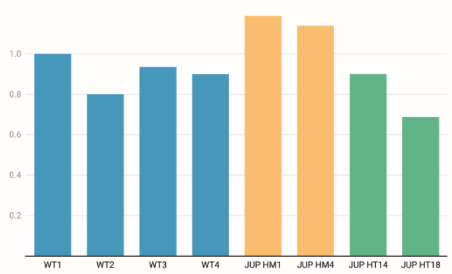

Figure S1. mRNA expression levels of edited genes. A) PKP2 levels in HM PKP2-PTC clones. B) DSG2 levels in DSG2-PTC clones. C) DSP levels in HM and HT DSP-PTC. D) JUP levels in HM and HT JUP-PTC.

Table S1. p value from qPCR results in HM DSP-PTC and HM JUP-PTC clones.

Gene	p value HM DSP-PTC	p value HM JUP-PTC
**PKP2**	0.4	0.975
**DSC2**	0.106	0.642
**DSG2**	0.863	0.221
**DSP**	0.229	0.45
**JUP**	0.625	0.017
**RYR2**	0.846	0.74
**ANK2**	0.629	0.017
**SLC8A1**	0.19	0.777
**ATP2A2**	0.629	0.652
**TRDN**	0.283	0.788
**CX43**	1	0.283
**NAV1.5**	0.8	0.871
**CASQ2**	0.052	0.687

Table S2. RQ values from qPCR results in HM JUP-PTC clones.

PG	PKP2	ANK2	ATPA2A	CASQ2	CX43	DSC2	DSG2	DSP	NAV1.5	PG	RYR2	SLC8A1	TRDN
**WT9**	1.00	1.00	1.00	1.00	1.00	1.00	1.00	1.00	1.00	1.00	1.00	1.00	1.00
**WT88**	0.84	0.64	0.27	0.91	0.52	0.42	0.80	0.79	0.71	0.80	0.55	0.56	0.71
**WT97**	1.42	0.81	0.41	1.08	0.69	0.87	1.33	1.62	1.35	0.94	0.85	0.95	0.73
**WT140**	1.06	0.93	0.62	1.36	1.12	1.21	1.70	1.25	1.47	0.90	1.04	0.81	0.80
**PG1**	1.04	1.42	0.57	1.37	0.69	1.48	0.89	0.98	1.60	1.19	0.98	0.97	0.91
**PG4**	1.14	1.26	0.33	0.97	0.43	0.63	0.64	0.90	0.53	1.14	0.59	0.58	0.77

Table S3. RQ values from qPCR results in HM DSP-PTC.

DSP	PKP2	ANK2	ATPA2A	CASQ2	CX43	DSC2	DSG2	DSP	NAV1.5	PG	RYR2	SLC8A1	TRDN
**WT9**	1	1	1	1	1	1	1	1	1	1	1	1	1
**WT88**	0.863	0.683	0.548	1,126	0.336	0.384	0.54	0.663	1,807	0.365	0.366	1.32	0.919
**WT97**	1,738	1,306	0.923	1,733	0.175	0.412	0.345	0.576	2,302	0.244	0.258	1.69	1,367
**WT140**	0.895	0.843	0.991	1,225	0.738	0.848	1	0.919	2,745	0.564	0.508	1,374	1,225
**DSP14**	0.747	1.625	0.955	0.878	0.406	0.407	0.545	0.381	1.037	0.682	0.314	1.006	1.272
**DSP15**	1.793	1.737	0.525	0.439	0.475	0.496	0.723	1.046	0.870	1.223	0.274	0.535	0.894
**DSP21**	0.791	0.511	0.623	0.736	0.406	0.001	0.644	0.370	0.363	0.517	0.567	0.809	0.723
**DSP28**	1,185	0.522	0.611	0.712	0.369	0.33	0.87	0.825	1,238	0.75	0.591	1,293	0.653

Table S4. Primers, PCR conditions and kits for Sanger sequencing

	**Primer sequence**	**PCR program**	**PCR Kit**
PKP2_FW	CAGTGTAGATTGGCACGTTTGT	PCR60	Gotaq (M743A, Promega)
PKP2_RV	GGGAAAGGAGACCACTTGAGA		
DSP_FW	TGAGCGGCTCTCTTG	Touchdown	Hotstart buff A + enhancer + DMSO (KK5515, KAPA biosystems)
DSP_RV	CCTGCGCTTCCGTGACC		
DSG2_FW	GGACCTGCCCAGGAGGAT	Touchdown	Hotstart buff GC (KK5515, KAPA biosystems)
DSG2_RV	TGCCTACCCTGCTCTCCC		
mDSC2_FW	AGTGGCCGATTCGAGTCTTT	PCR60	Gotaq (M743A, Promega)
mDSC2_RV	TCGCGCGCCTATTACAGCT		
mJUP_FW	GCTCCAGGAGAAGGGTAGAT	PCR59	Gotaq (M743A, Promega)
mJUP_RV	CGTGTCCTCAAGAGTGGACA		
